# Impacts of *Lysinibacillus sphaericus* on mosquito larval community composition and larval competition between *Culex pipiens* and *Aedes albopictus*

**DOI:** 10.1038/s41598-022-21939-1

**Published:** 2022-10-26

**Authors:** Joseph R. McMillan, Michael M. Olson, Tanya Petruff, John J. Shepard, Philip M. Armstrong

**Affiliations:** 1 Department of Biological Sciences, Texas Tech University, 2901 Main St., Rm 212, Lubbock, TX 79409 USA; 2 Department of Entomology, The Connecticut Agricultural Experiment Station, New Haven, CT USA

**Keywords:** Ecology, Diseases

## Abstract

Effectiveness of mosquito larvicide active ingredients (AI), such as *Lysinibacillus sphaericus*, varies between species, yet little is known regarding how differential effectiveness manifests in larval communities in applied settings. To examine how differential effectiveness of *L. sphaericus* influences larval community dynamics, we performed two experiments. We performed a field experiment in which containers were seeded with a standardized nutrient treatment, mosquitoes colonized the containers, and then containers received one of three *L. sphaericus* applications. We then performed competition assays between *Culex pipiens* and *Aedes albopictus* in low nutrient environments using multiple interspecific ratios and the presence/absence of a low dose of *L. sphaericus*. Field results demonstrated elimination of *Culex* spp. from treated containers while container breeding *Aedes* spp. proliferated across all treatments. *Lysinibacillus sphaericus* did not influence competition between *Cx. pipiens* and *Ae. albopictus*, and the *L. sphaericus* application eliminated *Cx. pipiens* in all treatment replicates while survival of *Ae. albopictus* was similar between treated and untreated containers across interspecific ratios. *Lysinibacillus sphaericus* is an effective AI for control of *Culex* spp. However, different AIs should be utilized in habitats containing non-*Culex* genera while a mix of AIs should be utilized where coexistence of multiple genera is expected or confirmed.

## Introduction

Biopesticide larvicide products are applied widely throughout the US to control nuisance and disease-transmitting mosquito vectors^1,2^. These larvicides, which vary in composition and concentration of their active ingredient (AI), are mostly derived from bacteria, are highly effective at controlling mosquito larval populations, and display minimal non-target effects on other aquatic insects and vertebrates^2^. The effectiveness of these products in the field depends on multiple factors including the conditions of the target environment and the bionomics of the target species. Recent interest in larvicide resistance as part of a broader pesticide resistance monitoring emphasis in the US has generated reports documenting variable mortality rates of different AIs among different species^3^ and between different populations of the same species. In particular, larval mortality stemming from exposure to the AI *Lysinibacillus sphaericus,* varies considerably among mosquito species^2^. Notably, *Culex pipiens* Linnaeus mosquito larvae are very susceptible to *L. sphaericus* while container breeding *Aedes* spp. such as *Aedes albopictus* [Skuse] are less susceptible^2,3^. Variable efficacy of *L. sphaericus* among different species may interfere with visual assessments of a treatment’s effectiveness—if the detection of larval or pupal individuals is the primary means of assessing effectiveness, then operational “failures” of detecting larvae in a treated environment could be attributable to detecting species tolerant of the AI.

Research with adulticides shows that certain products can alter or reverse asymmetric competitive outcomes in larval communities^4^; in some cases, single-species population reductions using pesticides can result in non-target species population expansions (most likely through the mechanism of competitive release)^5^. There are fewer investigations of the effect of larvicides on community composition and interspecific interactions. In terms of *Cx. pipiens* and *Ae. albopictus*, many studies document a general dichotomy between these two species’ larval habitats: *Cx. pipiens* larvae are typically found in eutrophic bodies of water such as those in catch basins^6–8^ while *Ae. albopictus* larvae are typically found in smaller, often cryptic, water sources^9,10^. Nevertheless, spatiotemporal co-occurrence of each species in the same habitats does occur^11^, which has generated interest in the dynamics of interspecific competition between the two species. Prior experiments have examined competitive outcomes as functions of nutrient availability^12–14^ and temperature gradients^15^, with asymmetric competitive advantages more commonly reported for *Ae. albopictus.*

Considering *L. sphaericus* products are widely used throughout the US for larval control in a variety of different habitats and sensitivity to this AI varies widely among species, these treatments may alter the structure and dynamics of mosquito communities, especially in environments that receive sub-lethal concentrations or when product effectiveness wanes post-application. The objectives of our study were twofold: (1) determine how varying concentrations of *L. sphaericus* alter mosquito larval community structure and assembly in treated semi-natural mesocosms, and (2) determine how sublethal concentrations of *L. sphaericus* alter interspecific interactions between *Cx. pipiens* and *Ae. albopictus* in laboratory competition assays. Our overall hypothesis was that *L. sphaericus* treatments alter the structure of larval communities in treated environments towards a greater proportional dominance of *L. sphaericus* tolerant container-breeding species such as *Ae. albopictus*, thereby providing a competitive advantage to these medically important vector species in the treated habitat.

## Results

### Project 1: mesocosm field experiments

**Figure 1:**
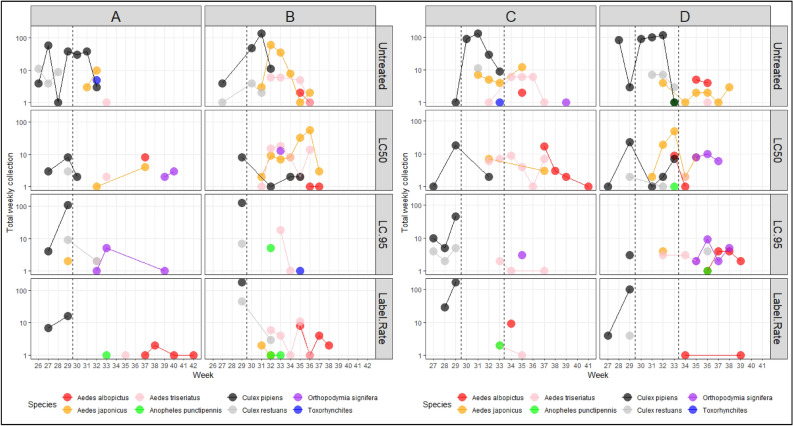
Total weekly produced adult mosquitoes in experimental mesocosms. All clusters contained treatment mesocosms which were initially treated with *Lysinibacillus sphaericus* (applied as VectoLex WDG) between weeks 29 and 30 (thin dashed line); a second application of *L. sphaericus* took place in Clusters C and D between weeks 33 and 34. Columns identify the cluster; rows identify the *L. sphaericus* treatment. Note that the y-axis is on a log10-scale. Figure was created using a combination of the R packages *ggpot2* and *tidyverse*.

Eight mosquito species colonized the mesocosms, including *Ae. albopictus* (n = 99), *Aedes japonicus* [Theobald] (n = 388), *Aedes triseriatus* [Say] (n = 196), *Anopheles punctipennis* [Say] (n = 13), *Cx. pipiens* (n = 1,896), *Culex restuans* Theobald (n = 151), *Orthopodomyia signifera* [Coquillett] (n = 71)*,* and *Toxorhynchites rutilus septentrionalis* [Dyar and Knab] (n = 7). Six species were found in each cluster across all treatment mesocosms, while *Or. signifera* pupae were not found in any of the Label Rate mesocosms and *Toxorhynchites* pupae were not found in any mesocosm in Cluster D. Visual presence of *Toxorhynchites* larvae was qualitatively documented in each cluster throughout the experiment, however, not counted. *Toxorhynchites* larval presence was first noted in a single mesocosm in Cluster A in Week 31 (~ August 5th); *Toxorhynchites* larval colonization of all mesocosms in Clusters A and B was noted in Week 38 (~ September 20th); and, the absence of pupae of any mosquito species was noted in association with the presence of *Toxorhynchites* larvae in all Clusters in Week 39 (~ September 27th). Figure 1 visually confirms the general absence of collected pupae of any species after Week 39.

There were more total mosquitoes collected in the untreated mesocosms across all clusters compared to the three other treatment levels. This result was due to the high abundance of *Cx. pipiens* pupae collected in the pre-treatment period of the experiment (Fig. 1, Table 1). Accounting for the presence of *Culex* spp. mosquitoes, post-hoc comparisons determined that more mosquitoes were collected in cluster B compared to all other clusters while mosquito collections were similar between the LC50, LC95, and Label rate mesocosms (Table 1, S. Fig. 1). When excluding *Culex* spp. mosquitoes from any analysis, more mosquitoes were found in the LC50 treated mesocosms compared to the untreated reference (Table 1, S. Fig. 1). Overall, there was a clear colonization pattern among species, with *Cx. pipiens* and *Cx. restuans* initially colonizing all mesocosms followed by the container *Aedes* spp. (Fig. 1). For all mesocosm clusters, *Culex* egg rafts were observed during sample collections throughout the course of the experiment.

**Table 1:** Summary results of a Poisson-error distributed generalized linear mixed effects model comparing total pupal collections in replicate mesocosms by applied treatment and cluster ID as fixed effects and week of collection and species identification as random effects.

Variable		With *Culex* spp.				Without *Culex* spp.			
		Est.	Std. error	z-value	Pr( >|z|)	Est.	Std. error	z-value	Pr( >|z|)
Intercept		− 2.25	0.80	− 2.83	0.005	− 4.74	0.93	− 5.1	3.5e−7
Cluster (reference A)	B	0.84	0.06	14.4	< 2e−16	1.93	0.14	13.7	< 2e−16
	C	0.50	0.06	8.16	3.5e−16	0.90	0.16	5.76	8.5e−9
	D	0.57	0.06	9.41	< 2e−16	1.19	0.15	7.89	3.0e−15
Treatment (reference untreated)	LC50	− 0.99	0.05	− 18.6	< 2e−16	0.56	0.08	6.73	1.7e−11
	LC95	− 1.13	0.06	− 20.2	< 2e−16	− 0.98	0.13	− 7.78	7.5e−15
	Label Rate	− 0.74	0.05	− 15.2	< 2e−16	− 1.24	0.14	− 8.90	< 2e−16
Random: week		Var = 4.51, Std. Dev = 2.13				Var = 7.7, Std. Dev = 2.77			
Random: species		Var = 2.93, Std. Dev = 1.71				Var = 2.17, Std. Dev = 1.48			

**Figure 2:**
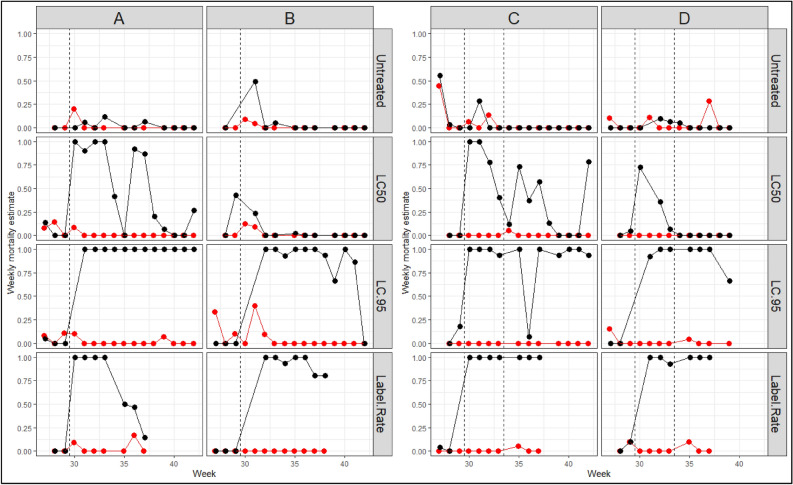
Weekly larval mortality estimates in bioassays of experimental mesocosm waters. All clusters contained treatment mesocosms which were initially treated with *Lysinibacillus sphaericus* (applied as VectoLex WDG) between weeks 29 and 30 (thin dashed line); a second application of *L. sphaericus* took place in Clusters C and D between weeks 33 and 34. Columns identify the cluster; rows identify the *L. sphaericus* treatment. Colors represent species: black—*Culex pipiens*; red—*Aedes albopictus*. Figure was created using a combination of the R packages *ggpot2* and *tidyverse*.

**Figure 3:**
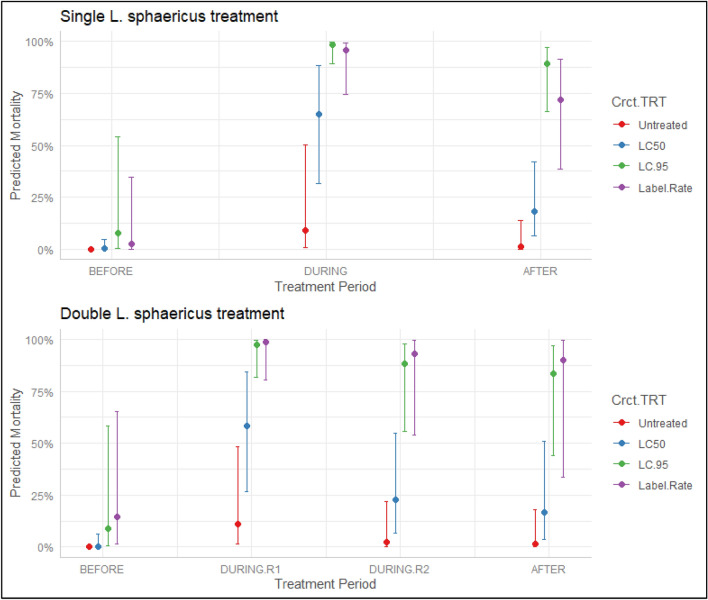
Predicted larval mortality of *Culex* spp. in water samples obtained from the experimental mesocosms. Predictions were generated from a binomial-error distributed generalized linear model with *Lysinibacillus sphaericus* concentration and treatment period as fixed effects. All clusters contained treatment mesocosms which were initially treated with *L. sphaericus* (applied as VectoLex WDG) between weeks 29 and 30; a second application of *L. sphaericus* took place in Clusters C and D between weeks 33 and 34. Points identify the average prediction holding all other variables constant, lines represent the 95% CI of the prediction, and colors identify the *L. sphaericus* treatment. Figure was created using a combination of the R packages *ggeffects, ggpot2* and *tidyverse*.

Weekly mortality assays using mesocosm water and larvae from the Connecticut Agricultural Experiment Station’s (CAES) *Cx. pipiens* and *Ae. albopictus* colonies confirmed three observations from the field experiment: (1) The LC95 and Label Rate applications of *L. sphaericus* remained effective at killing *Cx. pipiens* larvae throughout the duration of the experiment (Fig. 2), (2) the LC50 treatment showed less residual activity compared to the LC95 and Label Rate treatments (Table 2, Fig. 3), and (3) *L. sphaericus* exposure had little to no mortality effect on the development and survival of *Ae. albopictus* larvae (Fig. 2). Generated susceptibility curves for each species confirmed *Ae. albopictus* was tolerant of *L. sphaericus* (S. Fig. 2).

**Table 2:** Summary results of a binomial-error distributed generalized linear model comparing *Culex pipiens* mortality during a single or double treatment regimen of multiple *L. sphaericus* concentrations from bioassays of mesocosm water samples.

Variable		Single *L. sphaericus* treatment				Double *L. sphaericus* treatment			
		Est.	Std. error	z-value	Pr( >|z|)	Est.	Std. error	z-value	Pr( >|z|)
Intercept		− 9.02	1.95	− 4.62	3.8e−6	− 8.02	1.75	− 4.58	4.7e−6
Treatment (reference untreated)	LC50	2.95	1.32	2.24	0.03	2.44	1.14	2.14	0.032
	LC95	6.57	1.52	4.33	1.5e−5	5.68	1.35	4.20	2.7e−5
	Label	5.41	1.48	3.65	0.00026	6.26	1.64	3.82	0.00014
Treatment period (reference before)	DURING	6.69	1.64	4.08	4.6e−5	5.92	1.54	3.85	0.00012
						4.36	1.42	3.08	0.0021
	AFTER	4.56	1.44	3.17	0.0015	3.96	1.54	2.56	0.01

### Project 2: laboratory competition assays

**Figure 4:**
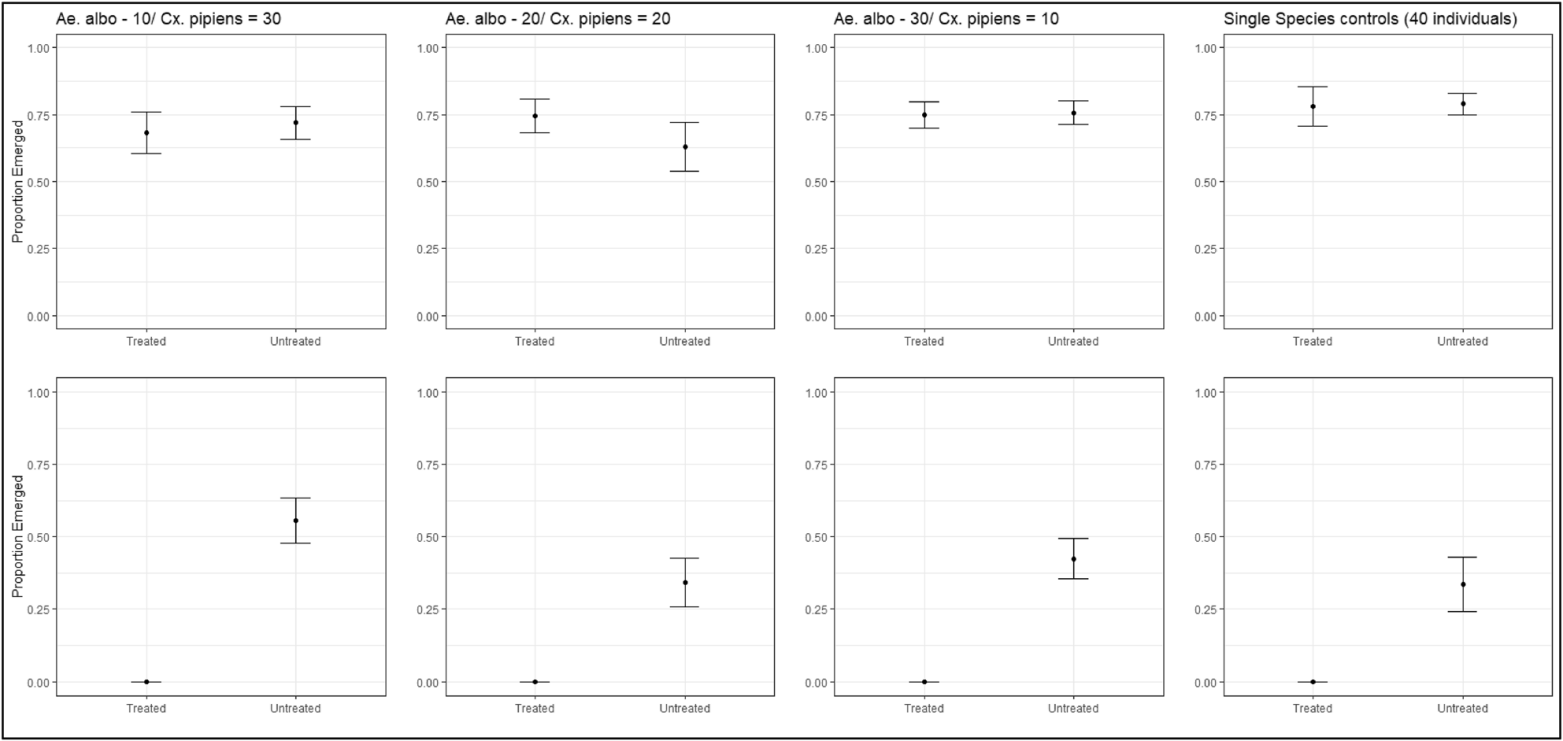
Average (+/− SE) survival to emergence of *Aedes albopictus* (top rows) and *Culex pipiens* (bottom row) in experimental trials in untreated and treated containers (0.01 ITU/ml *Lysinibacillus* sphaericus). All containers were initiated with 120 mg of a 3:2 liver powder and baker’s yeast mixture. Experiment 1 was initiated with the addition of 1 ITU *L. sphaericus* and 40 1st instar larvae and columns indicate the number of those individuals as *Ae. albopictus* and *Cx. pipiens*. Figure was created using a combination of the R packages *ggpot2* and *tidyverse*.

In experiment 1, we evaluated whether *L. sphaericus* applications could increase the survival of a non-target species (*Ae. albopictus*) by releasing them from competition from *Cx. pipiens* larvae. The LC25 application of *L. sphaericus* resulted in 100% mortality of all *Cx. pipiens* larvae, and there was no effect of *L. sphaericus* on *Ae. albopictus* survival compared to the untreated containers. Additionally, the increased availability of nutrients to *Ae. albopictus* in the LC25 treated cups did not significantly increase survival to pupation compared to the untreated controls (Fig. 4). When only examining the untreated cups, *Ae. albopictus* survival was greater than *Cx. pipiens* across all replicate interspecific ratios (Fig. 4). Additionally in the untreated cups, there were no significant differences in species-specific survival among the respective replicate interspecific ratios (Table 3).

**Table 3:** Summary results of a binomial-error distributed generalized linear model comparing species-specific survival in untreated replicate containers from experiment 1.

Variable		*Culex pipiens*				*Aedes albopictus*			
		Est.	Std. error	z-value	Pr( >|z|)	Est.	Std. error	z-value	Pr( >|z|)
Intercept		0.08	0.52	0.16	0.88	0.94	0.58	1.64	0.1
Interspecific ratio (reference 10/30)	20/20	− 0.24	0.73	− 0.33	0.74	− 0.32	0.79	− 0.4	0.69
	30/10	0.37	0.74	0.50	0.62	0.08	0.83	0.10	0.92
	40/0	− 0.76	0.87	− 0.87	0.38	0.38	1.00	0.38	0.70

All containers were initiated with 120 mg of a 3:2 liver powder and baker’s yeast mixture. Experiment 1 consisted of the addition of 1 ITU *L. sphaericus* and 40 1st instar larvae.

In experiment 2, we assessed the combined impacts of nutrient depletion by *Cx. pipiens* larvae and *L. sphaericus* toxicity on *Ae. albopictus* colonization. When *Culex pipiens* larvae were allowed to develop and pupate and then the containers were treated with the *L. sphaericus* LC25 and *Ae. albopictus* were added, there was no effect of reduced nutrient availability and larvicide presence on *Ae. albopictus* survival to pupation (Condition: F = 2.9, p = 0.09; *L. sphaericus* treatment: F = 0.01, p = 0.92) (S. Fig. 3). There were also no differences in survival to pupation between *Cx. pipiens* and *Ae. albopictus* nor among *Cx. pipiens* replicate ratios (S. Fig. 3).

## Discussion

Our combined results from semi-natural mesocosms, susceptibility curves, mortality assays, and competition assays confirm variable mortality rates in *L. sphaericus* exposed *Cx. pipiens* and *Ae. albopictus* larvae. Overall, LC95 and Label Rate treatments of *L. sphaericus* immediately removed *Cx. pipiens* and *Cx. restuans* from our treatment mesocosms; sustained removal of *Cx. pipiens* from developing in these environments is supported by our mortality assays showing long periods of the AI’s residual activity. Additionally, our field experiments demonstrate that *Aedes* spp. mosquitoes readily colonize habitats treated for *Cx. pipiens* larvae; mortality assays with *Ae. albopictus* larvae further support the ability of container *Aedes* spp. to successfully develop into adults despite the presence of *L. sphaericus*. We were unable to demonstrate evidence of larvicide-mediated competition between *Cx. pipiens* and *Ae. albopictus.* While *Ae. albopictus* development rates were greater than *Cx. pipiens* in the untreated replicates, there was no difference in *Ae. albopictus* development between treated and untreated containers; this was despite the total elimination of *Cx. pipiens* larvae in the treated replicates. In all, our results clearly demonstrate the utility of *L. sphaericus* for effective control of *Cx. pipiens* larvae. However, other product formulations and AIs should be used for control of container breeding *Aedes* spp.

Though our results provide a proof-of-principle analysis of differential mortality rates in *L. sphaericus* exposed *Cx. pipiens* and *Ae. albopictus* larvae, our field results were unable to control for all factors that influence community composition. For instance, changes in nutrient concentrations and availability, as well as predation, are factors that deserve attention. All replicates were initially seeded with the same amount of nutrients, and qualitatively, these containers initially resembled solutions used to bait CDC gravid traps: containers were visually murky and smelled eutrophic. However, we did not prevent rainfall or leaf litter from entering (nor did we remove from) containers, and no additional nutrients were added throughout the experiment. The combination of these factors likely influenced the microbial and nutritional composition in our containers, leading to a qualitative change in each mesocosm’s aquatic environment, i.e., water became clearer and there was a general absence of a smell. Changes in microbial and/or nutritional composition could have been an important explanatory variable in the transition from *Culex* spp. to *Aedes* spp. dominance in the untreated and LC50 containers after around 8 weeks. However, the detection of *Culex* egg rafts in all mesocosms throughout the length of the experiment suggests mesocosms were suitable oviposition sites regardless of the absence of collected pupae in later weeks that could have been associated with water quality differences between pre- and post-treatment periods. Prior research has also shown an inhibitory effect of *Aedes* spp. presence on *Culex* larval abundance in semi-natural experiments^16^, indicating that colonization of our mesocosms by *Aedes* spp. further limited the development of *Culex* individuals in the untreated and LC50 treatment containers. Based on comparisons to the LC95 and Label Rate mesocosms, our treatments at most accelerated this community succession due to the toxicity of the treated environment to *Culex* spp. It is important to note that these observed changes in water quality were only noted during the experiment and follow up experiments should track changes in chemicals such as ammonia, nitrate, and phosphate, which are all indicators of nutrient richness^17^, or define changes in microbial composition using genomics approaches. Future experiments should also consider the addition of nutrients at different intervals to better isolate the hypothesis that *L. sphaericus* drives changes in larval community structure.

An additional complication of our field experiment was the unexpected colonization of each container by larvae of the genus *Toxorhynchites. Toxorhynchites* is an important predator of container developing mosquito larvae, and previous experiments show that predation by *Toxorhynchites* can result in significant alterations of mosquito community composition^18,19^. The strength of *Toxorhynchites* predation effects on mosquito larval survival and development is the driving reason behind mass rearing and release programs of *Toxorhynchites* mosquitoes as a biological control tactic^20,21^. Because *Toxorhynchites* pupae were not detected in a single mesocosm until the 8th week of our experiment, our results confidently demonstrate that the application and continued residual activity of *L. sphaericus* was dominantly responsible for the absence of *Cx. pipiens* larval development in the post-treatment periods. Beyond this initial application phase, we cannot fully conclude that sublethal effects of *L. sphaericus* resulted in community composition changes. Like the consideration of nutrients, follow up experiments should include a *Toxorhynchites* removal treatment to better isolate the hypothesis that L. *sphaericus* drives changes in larval community structure.

Prior studies of interspecific competition between *Cx. pipiens* and *Ae. albopictus* on average report asymmetric outcomes in which *Ae. albopictus* is the dominant competitor^12–15^. Our results from Experiment 1 support these findings and show that *Ae. albopictus* development rates were greater than *Cx. pipiens* rates in the untreated replicates. The rebound in *Cx. pipiens* survival in the absence of *Ae. albopictus* in Experiment 2 provides further evidence of asymmetric competition between the two species. Nutrient availability, which in Experiment 2 was not based on the number of larvae initially seeded into the replicate as in Experiment 1, could explain differential survival of *Cx. pipiens* between Experiment 1 and 2; however, there was no difference in *Cx. pipiens* survival across the three nutrient levels in Experiment 2 suggesting the presence of *Ae. albopictus* was the dominant factor explaining reduced survival of *Cx. pipiens* in the untreated replicates in Experiment 1. Given the demonstrated specificity of *L. sphaericus* for control of *Cx. pipiens*, other AIs should be considered in future studies of insecticide-mediated competition between these two important vector species.

## Conclusion

Larvicides are an important part of any Integrated Vector Management tool kit. Our research shows that certain active ingredients, such as *Lysinibacillus sphaericus* (the AI of VectoLex), are effective in a limited number of species. We were able to demonstrate residual mortality of *L. sphaericus* in field and lab exposed *Cx. pipiens* mosquitoes while other mosquito species, mostly container breeding *Aedes* spp., were able to colonize and successfully develop in treated habitats. Target mosquito species and method of larvicide effectiveness evaluations should be strongly considered when planning an anti-vector intervention campaign.

## Methods

### Project 1: mesocosm field experiments

Mesocosm experiments took place at Lockwood Farm located in Hamden, Connecticut. Individual mesocosms were composed of black 20 L cylindrical plastic containers filled with 12 L tap water and seeded with 10 mg of a 3:2 ratio liver powder/brewer’s yeast mixture and 1 g of grass hay. Drain-holes were drilled into the sides of each container 5 mm from the 12 L surface to allow flooding for *Aedes* spp. egg emergence and to allow overflow beyond this level due to precipitation. Four experimental mesocosm clusters were dispersed throughout the Lockwood Farm in microhabitats previously sampled in Eastwood et al.^22^. Clusters contained 4 mesocosms spaced 3 m apart in a 2 × 2 grid. We utilized four *L. sphaericus* treatment levels in each cluster: no *L. sphaericus*, the LC50 (0.053 ITU/ml) and LC95 (1.0 ITU/ml) for *Culex pipiens* derived from Burtis et al.^3^, and the label rate of *L. sphaericus* (~ 1.2 ITU/ml). All treatments were derived from VectoLex WDG. Prior to insecticide application, we prepared 1 L of a 1000 ITU/ml stock solution. To inoculate each mesocosm, we measured the depth of the container’s water column, calculated water volume, and applied the appropriate amount of stock to achieve the target LC value. Replicate insecticide treatments were randomized within each cluster, and insecticides were applied 30-days post mesocosm seeding with nutrients. All mesocosms in each cluster were rotated within the 2 × 2 grid each week. Two clusters were then randomly chosen for a second application of *L. sphaericus* 30-days post initial insecticide application.

To sample the larval habitat of each mesocosm, we performed a figure-8 sweep with an aquarium fish net (4 × 3-in. opening, Penn-Plax) each Monday and Thursday of the week for each week of the experiment. Sweep contents were washed from the net into a white photo development pan, and pupae were removed for in-lab identification after eclosion following a dichotomous key^23^. All larvae were then returned to the mesocosm. This sampling protocol minimized destruction of larval habitats and influence of interspecific interactions due to removal sampling.

In addition to sampling containers for pupae, we collected water samples from each container for an in-lab bioassay to determine the realized mortality of the larval environment. Due to time constraints of the field crew, a 50% randomized sample of containers were sampled on Monday with the remaining 50% sampled on Thursday of each sampling week. Bioassay procedures followed McMillan et al.^24^ for *Cx. pipiens* with the addition of screening mortality in CAES’ *Ae. albopictus* colonies. We finally performed in-lab susceptibility trials to *L. sphaericus* with larvae from CAES’ *Cx. pipiens* and *Ae. albopictus* colonies to confirm each species’ colony varied in their sensitivity to the product. Briefly, 15 3rd to 4th instar larvae of each species per replicate dose were exposed to a wide range of *L. sphaericus* concentrations and mortality was recorded 24-h post-exposure. Lethal concentrations were then estimated from a generalized linear model with mortality (corrected for mortality in untreated control replicates) as the response term and the log_10_-dose as the predictor term.

Primary endpoints from the field experiment included the number and species identity of pupae collected from each mesocosm. We compared total weekly pupal collections per mesocosm using a generalized linear mixed model (GLMM) framework with treatment level and cluster ID as fixed effects, species ID and week of collection as a random effect, and a Poisson-error distribution. We repeated this analysis excluding all collected *Culex* spp. to examine how the *L. sphaericus* treatments impacted the more tolerant *Aedes* spp. The primary endpoint for the mortality assays was the corrected larval mortality. We initially compared mortality using a species-specific GLMM with *L. sphaericus* treatment concentration and treatment period as fixed effects, week of collection as a random effect, and a binomial-error distribution. Preliminary analyses revealed negligible variance attributed to week of collection, so all subsequent models were a GLM. All analyses were performed in R V4.1.3^25^ using the following packages: tidyverse^26^, gridExtra^27^, ggplot2^28^, ggeffects^29^, and glmmTMB^30^.

### Project 2: laboratory competition assays

Competition assays took place at CAES’ main facility in New Haven, CT. This facility contains an *Ae. albopictus* colony (founded circa 2014 from Stratford, CT) and a *Cx. pipiens* colony (founded circa 2018 from New Haven, CT;). Colony maintenance for each species was similar: larval rearing pans consisted of approx. 200 eggs (on papers, *Ae. albopictus*, or as egg rafts, *Cx. pipiens*) in ~ 2 L RO water and initiated with ~ 20 ml of a 1% 3:2 liver powder/brewer’s yeast slurry. Pans were held at 25.5 °C and 80% humidity and fed ~ 20 ml of the 1% slurry every other day. Pupae were removed to an eclosion chamber and adults were allowed access to 10% sucrose solution ad libitum. *Aedes albopictus* females were given access to defibrinated sheep’s blood (HemoStat©) through a Hemotek membrane feeder for 1 h every 2–3 weeks and moistened, fluted filter paper was provided to collect eggs. *Culex pipiens* females were given access to a live, restrained buttonquail overnight once per week and a small cup seeded with 5 ml 1% slurry and 15 RO ml water was provided to collect egg rafts. The use of buttonquail was reviewed and approved in accordance with CAES Institutional Animal Care and Use Committee.

We performed two experiments. All experiments consisted of the following treatments: variable ratios of *Ae:Cx* larvae and two *L. sphaericus* treatments (no treatment and 0.01 ITU/ml). Larval density (40 per container) remained constant across all replicate treatments, but *Ae:Cx* ratios varied from 40/0, 30/10, 20/20, 10/30, and 0/40. Nutrients supplied were a low concentration (3 mg larva^−1^) of a 3:2 liver powder/brewer’s yeast mix applied at the beginning of the experiment. Temperature was held constant at the colony maintenance level. Assays took place in 300 ml disposable plastic cups filled with 100 ml of RO water. The first experiments consisted of the addition of the 40 larvae as newly hatched individuals (+/− 1 day between species’ hatch) at the appropriate ratios, the larval diet, and the 0.01 ITU/ml concentration (diluted from a lab stock of 1000 ITU/ml). Assays were monitored daily until all larvae were dead and/or all larvae pupated. Experiment 2 consisted of the addition of only the *Cx. pipiens* larvae and the larval diet. After all *Cx. pipiens* had pupated, containers were treated with *L. sphaericus* and then the *Ae. Albopictus* larvae were added.

Primary endpoints included species-specific pupation success. Preliminary analyses in a GLMM framework revealed negligible variance attributed to a replicate ID random effect; replicate as a random term also interfered with model convergence. Preliminary analyses further revealed there was neither a significant interaction nor an improvement in the Akaike Information Criterion between the *L. sphaericus* treatment and initial starting condition terms. Thus, we adopted a GLM rather than a GLMM framework in all further analyses, and species-specific mortality was analyzed as a binomial response term with treatment and initial starting conditions included as fixed effects All analyses were performed in R V4.1.3^25^ using the following packages: tidyverse^26^, gridExtra^27^, and ggplot2^28^.

## Supplementary Information

Supplementary Figures.

Supplementary Information 2.

## Data Availability

All raw data is included as supporting information attached to the manuscript.

## References

[1] Regis L, Silva-Filha MH, Nielsen-LeRoux C, Charles JF (2001). Bacteriological larvicides of dipteran disease vectors. Trends Parasitol..

[2] Lacey LA (2007). *Bacillus thuringiensis* serovariety israelensis and *Bacillus sphaericus* for mosquito control. J. Am. Mosq. Control Assoc..

[3] Burtis JC (2021). NEVBD pesticide resistance monitoring network: Establishing a centralized network to increase regional capacity for pesticide resistance detection and monitoring. J. Med. Entomol..

[4] Alto BW, Lampman RL, Kesavaraju B, Muturi EJ (2013). Pesticide-induced release from competition among competing *Aedes aegypti* and *Aedes albopictus* (Diptera: Culicidae). J. Med. Entomol..

[5] Qureshi A, Connolly JB (2021). A systematic review assessing the potential for release of vector species from competition following insecticide-based population suppression of *Anopheles* species in Africa. Parasit. Vectors.

[6] Geery PR, Holub RE (1989). Seasonal abundance and control of *Culex* spp. in catch basins in Illinois. J. Am. Mosq. Control Assoc..

[7] Su T, Webb JP, Meyer RP, Mulla MS (2003). Spatial and temporal distribution of mosquitoes in underground storm drain systems in Orange County, California. J. Vector Ecol..

[8] Vazquez-Prokopec GM (2010). The risk of West Nile Virus infection is associated with combined sewer overflow streams in urban Atlanta, Georgia, USA. Environ. Health Perspect..

[9] Unlu I, Farajollahi A, Strickman D, Fonseca DM (2013). Crouching tiger, hidden trouble: Urban sources of *Aedes albopictus* (Diptera: Culicidae) refractory to source-reduction. PLoS ONE.

[10] Unlu I, Faraji A, Indelicato N, Fonseca DM (2014). The hidden world of Asian tiger mosquitoes: Immature *Aedes albopictus* (Skuse) dominate in rainwater corrugated extension spouts. Trans. R. Soc. Trop. Med. Hyg..

[11] Reiskind MH, Hopperstad KA (2017). Surveillance for immature mosquitoes in windshield wash basins at gas stations. J. Med. Entomol..

[12] Costanzo KS, Mormann K, Juliano SA (2005). Asymmetrical competition and patterns of abundance of *Aedes albopictus* and *Culex pipiens* (Diptera: Culicidae). J. Med. Entomol..

[13] Costanzo KS, Muturi EJ, Lampman RL, Alto BW (2011). The effects of resource type and ratio on competition with *Aedes albopictus* and *Culex pipiens* (Diptera:Culicidae). J. Med. Entomol..

[14] Muller R (2018). Larval superiority of *Culex pipiens* to *Aedes albopictus* in a replacement series experiment: Prospects for coexistence in Germany. Parasit. Vectors.

[15] Marini G (2017). The effect of interspecific competition on the temporal dynamics of *Aedes albopictus* and *Culex pipiens*. Parasit. Vectors.

[16] Murrell EG, Juliano SA (2013). Predation resistance does not trade off with competitive ability in early-colonizing mosquitoes. Oecologia.

[17] Lund A (2014). Long term impacts of combined sewer overflow remediation on water quality and population dynamics of *Culex **quinquefasciatus*, the main urban West Nile virus vector in Atlanta, GA. Environ. Res..

[18] Ellis AM, Lounibos LP, Holyoak M (2006). Evaluating the long-term metacommunity dynamics of tree hole mosquitoes. Ecology.

[19] Paradise CJ (2008). Local and regional factors influence the structure of treehole metacommunities. BMC Ecol..

[20] Donald CL, Siriyasatien P, Kohl A (2020). Toxorhynchites species: A review of current knowledge. Insects.

[21] Vinogradov DD, Sinev AY, Tiunov AV (2022). Predators as control agents of mosquito larvae in micro-reservoirs (review). Inland Water Biol..

[22] Eastwood G (2020). Evaluation of novel trapping lures for monitoring exotic and native container-inhabiting *Aedes* spp. (Diptera: Culicidae) mosquitoes. J. Med. Entomol..

[23] Andreadis TG, Thomas MC, Shepard JJ (2005). Identification guide to the mosquitoes of Connecticut. Conn. Agric. Exp. Stn. Bull..

[24] McMillan JR (2021). The community-wide effectiveness of municipal larval control programs for West Nile virus risk reduction in Connecticut, USA. Pest. Manag. Sci..

[25] R Development Core Team, R. *R: A language and environment for statistical computing* (R Foundation for Statistical Computing, 2008).

[26] Wickham, H. The tidyverse. *R package ver* **1**, 1 (2017).

[27] Auguie, B., Antonov, A. & Auguie, M. B. Package ‘gridExtra’. *Miscellaneous Functions for “Grid” Graphics* (2017).

[28] Wickham, H., Chang, W. & Wickham, M. H. Package ‘ggplot2’. *Create elegant data visualisations using the grammar of graphics. Version* **2**, 1–189 (2016).

[29] Lüdecke D (2018). ggeffects: Tidy data frames of marginal effects from regression models. J. Open Source Softw..

[30] Brooks ME (2017). glmmTMB balances speed and flexibility among packages for zero-inflated generalized linear mixed modeling. R. J..

