# Supplementary material for: Impacts of Lysinibacillus sphaericus on mosquito larval community composition and larval competition between Culex pipiens and Aedes albopictus
Source: Sci Rep. 2022 Oct 26;12:18013. doi: 10.1038/s41598-022-21939-1 (PMC9606275; doi:10.1038/s41598-022-21939-1)
Supplement: Supplementary file 1 — Supplementary Figures. [file 41598_2022_21939_MOESM1_ESM.docx]

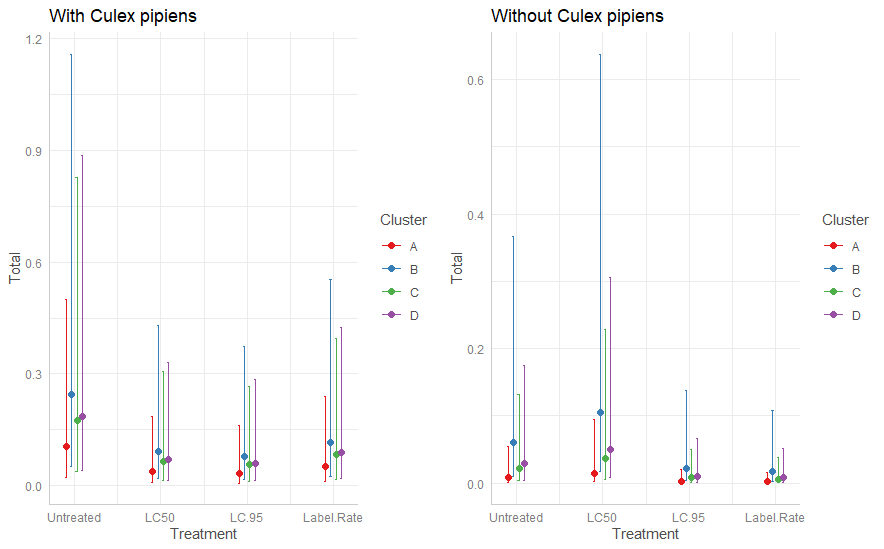


**S. Figure 1:** Predicted total weekly produced adult mosquitoes in experimental mesocosms. Predictions were generated from a Poisson-error distributed generalized linear mixed effects model with cluster ID and treatment as fixed effects and week of collection and species ID as crossed random effects. All clusters contained treatment mesocosms which were initially treated with *Lysinibacillus sphaericus* (applied as VectoLex WDG) between weeks 29 and 30 (thin dashed line); a second application of *L. sphaericus* took place in Clusters C and D between weeks 33 and 34. Points identify the average prediction holding all other variables constant, lines represent the 95% CI of the prediction, and colors identify the Cluster ID.


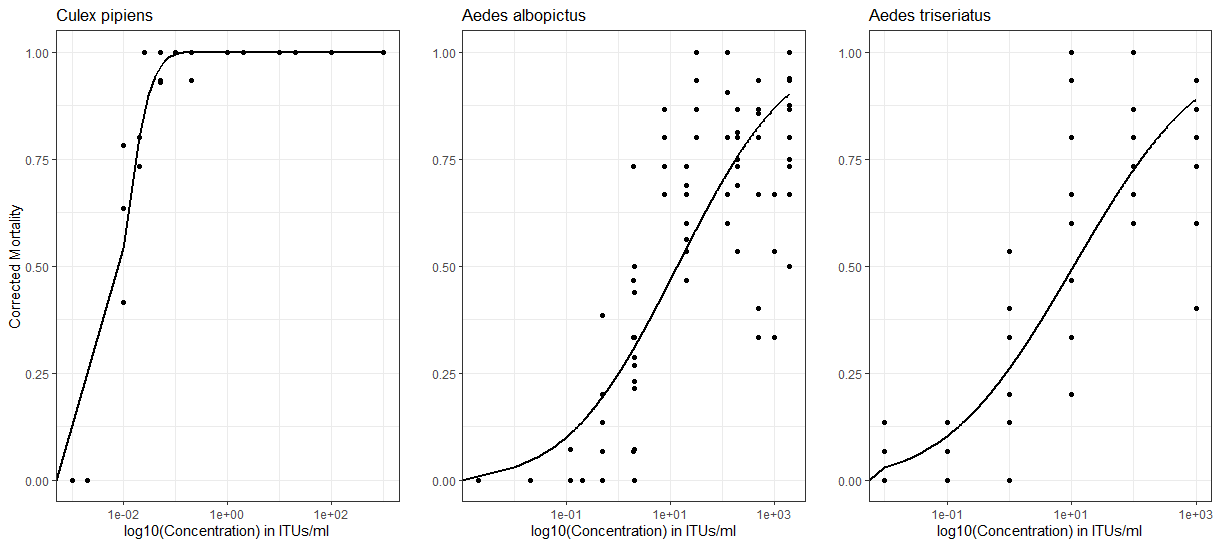


**S. Figure 2:** *Lyscinibaccilus sphaericus* susceptibility curves for *Culex pipiens* and *Aedes albopictus.* Points represent observed mortality (corrected for mortality in untreated containers) at each replicate dose; lines represent the predicted mortality from a quasi-poisson distributed generalized linear model with mortality of the response variable and dose as the predictor variable.


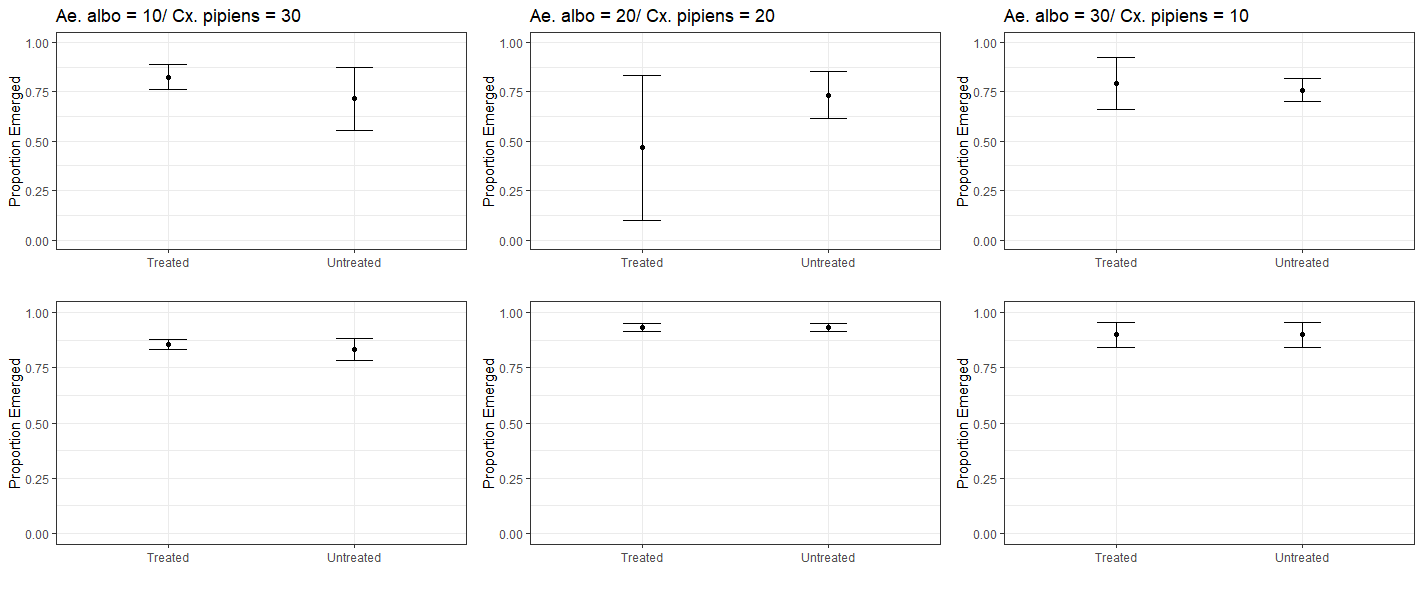


**S. Figure 3:** Average (+/- SE) survival to emergence of *Aedes albopictus* (top rows) and *Culex pipiens*(bottom row) in experimental trials in untreated and treated containers (0.01 ITU/ml VectoLex). All containers were initiated with 120 mg of a 3:2 liver powder and baker’s yeast mixture. Experiment 2 was initiated with the addition of the specific density of *Cx. pipiens* 1^st^ instar larvae which were then allowed to develop into pupae; after pupation, containers were either treated or not with the LC25 and the specific density of *Ae. albopictus* 1^st^ instar larvae were added. Columns indicate the number of those individuals as *Ae. albopictus*and *Cx. pipiens*.
